# Investigating migraine phenotype and dynamics in women with endometriosis: an observational pilot study

**DOI:** 10.1007/s13760-024-02484-2

**Published:** 2024-06-15

**Authors:** Gabriele Merki-Feld, Hanna Dietrich, Patrick Imesch, Andreas R. Gantenbein, Peter Sandor, Christoph J. Schankin

**Affiliations:** 1 Department of Reproductive Endocrinology, University Hospital Zurich, 8091 Zurich, Switzerland; 2 Department of Gynaecologic Endocrinology, University Hospital Zurich, Frauenklinikstrasse 26, 8091 Zurich, Switzerland; 3 Department of Gynaecology, University Hospital Zürich, Zurich, Switzerland; 4 Department of Neurology and Pain, ZURZACH Care, Bad Zurzach, Switzerland; 5 Neurologie am Untertor, Bülach, Switzerland; 6 Department of Neurology, Inselspital, Bern University Hospital, University of Bern, Bern, Switzerland

**Keywords:** Migraine, Endometriosis, Aura, Dysmenorrhea

## Abstract

**Introduction:**

Migraine and endometriosis are chronic disabling pain conditions. There is evidence for a shared genetic background. Migraine phenotype and course in patients with the comorbidity are insufficient investigated. Both conditions can be treated with progestins.

**Methods:**

For this observational study we included women with migraine and endometriosis, visiting our clinic from 2015 to 2021. We collected available information from charts and complemented these data by a structured phone interview to collect more specific information on migraine and the course of both diseases.

**Results:**

From 344 patients fulfilling the inclusion criteria, 94 suffered from both, endometriosis and migraine. Migraine with aura was reported by 41% of the patients and was associated with earlier onset of migraine (age < 17 years (OR 6.54) and with a history of medication overuse headache (OR 9.9, CI 1.6–59.4). Present monthly migraine frequency (1.5 ± 2.6) was significantly lower than five years before the interview (2.9 ± 4.64). There was a correlation between medication overuse headache and use of analgesics more than 3 days/months for dysmenorrhoea (*p* < 0.03). ASRM endometriosis score was not associated with migraine characteristics.

**Conclusions:**

We conclude that the comorbidity of endometriosis is highly linked to migraine with aura. Migraine onset in these patients was earlier. Further studies are needed to explore, if the observed decrease in migraine frequency can be attributed to recent endometriosis surgery and to understand if early diagnosis and treatment of both conditions may contribute to improve the course of both conditions.

*Trial registration* BASEC Nr. 2021-00285.

## Introduction

Both, endometriosis and migraine are chronic inflammatory disorders with estrogens playing a pivotal role in the pathophysiology [1–4]. Both conditions are associated with chronic pain and a high grade of disability in women during the reproductive years. Typically, symptoms decrease, when women approach menopause [5, 6]. Today there is high evidence that both conditions might share a common genetic background and that polymorphisms of sex hormones play a crucial role [3, 4, 7]. In a large case–control study, Yang et al. found among 20,220 patients with endometriosis a 1.7-fold higher prevalence of migraines than in controls.[8]. Survey-based case–control studies in endometriosis patients reported migraine in 29–69% of the affected women, which is far higher than the expected prevalence in the general female population [1, 2, 9]. Both conditions respond to treatment with the synthetic progestin desogestrel, which is used continuously and inhibits ovulation [10–15].

Severe dysmenorrhea without response to pain killers is one of the typical early symptoms in adolescents with endometriosis [16]. Early diagnosis of both conditions and specific treatment is highly relevant to reduce the probability of developing chronic pelvic pain and possibly corresponding alterations of pain response in the brain [17]. Women with comorbid migraine and endometriosis might be at increased risk for medication overuse headache (MOH), as they need to use painkillers for menstrual and non-menstrual pelvic pain and in addition for their headaches. While several studies investigated the prevalence of migraine in women with endometriosis, there is only minimal knowledge on migraine characteristics in women with the comorbidity. Theoretically, endometriosis patients might be more prone to suffer from chronic migraine or a higher migraine frequency. It is unknown if women with this comorbidity suffer rather from migraine with aura (MA). There seems to be a genetic component [18]. Women during the reproductive years tend to suffer more from menstrual migraine without aura (MO). With the present study, we aimed to identify migraine phenotypes in women with migraine and endometriosis and the dynamics of migraine in these patients. In particular we focused on aura, monthly migraine frequency (MMF) at present during puberty and 5 years ago, age at migraine onset, history of medication overuse headache, use of prophylactic agents and quality of life were in our focus.

## Materials and methods

### Study design

This observational study was conducted at the Clinic for Reproductive Endocrinology in the Department of Gynaecology of the University Hospital of Zurich. Data were collected from patient records and supplemented through telephone interviews. The presented data is part of a broader study investigating the characteristics of endometriosis in women with different comorbidities.

### Data collection

**Fig. 1:**
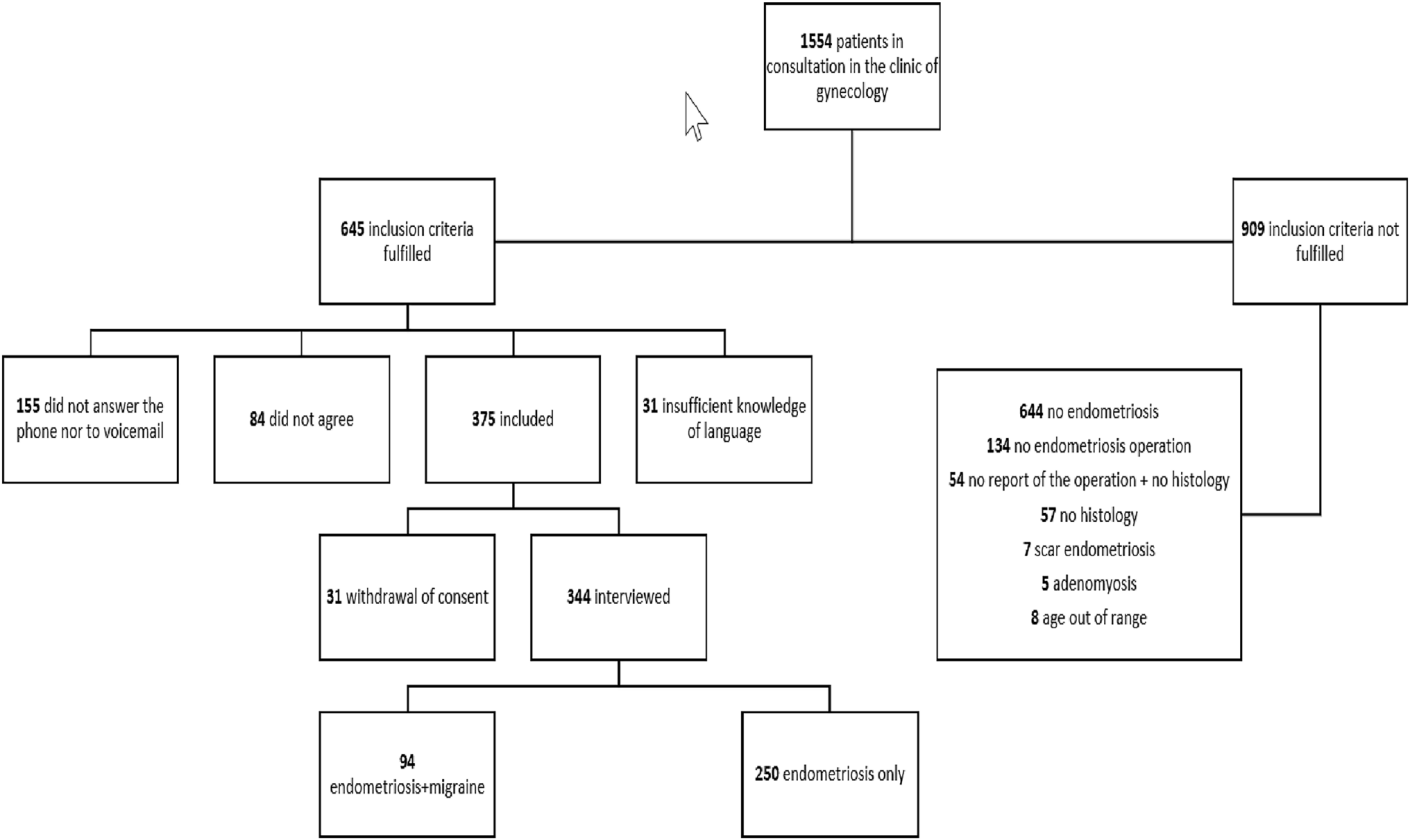
The flowchart containing the recruiting, inclusion and data collection process

Potential participants with the diagnosis of endometriosis and migraine were preselected by searching charts from all endometriosis patients treated in our outpatient clinic from January 2015 to July 2021. Included were all premenopausal patients aged > 18 years with biopsy-confirmed endometriosis, who in addition suffered from migraine (Fig. 1). Diagnosis of migraine and migraine with aura were evaluated in telephone interviews using the criteria of the International Classification of Headache Disorders (3rd version, ICHD-3) of the International Headache Society to ensure the correct differentiation between migraine and other types of headaches [19]. In order to increase reliability, both interviewers were trained with 50 interviews prior to start of the study. Postmenopausal women and those with adenomyosis or scar endometriosis were excluded. During a phone contact, we informed patients about the study and asked if they were interested in participating. Those willing to participate received an information sheet and provided consent. Details about the inclusion process are presented in Fig. 1. The interview questions were adapted from a structured questionnaire developed from the World Endometriosis Research Foundation “Women’s Health Symptom Survey Questionnaire” [20]. The questionnaire covers demographics, height, weight, medical conditions, operations, family history, menstruation, pregnancies, deliveries, potential symptoms of endometriosis, and medication. In addition 30 headache-specific questions, were added, including age at migraine onset, migraine frequency in the past (at age < 20 years and 5 years ago) and at present, aura, triggers, acute and prophylactic medication and disability, using the Migraine Disability Assessment questionnaire (MIDAS) [21]. From the surgery reports, we had access to information about endometriosis score (ASRM), infiltration depth, number of affected compartments, and localization [22].

### Statistical analyses

We performed all statistical analyses using IBM SPSS version 27.0.1.0. We presented categorical variables as frequencies and continuous variables as means with standard deviations. For comparison of subgroups we calculated independent sample *t*-tests for normally distributed variables and Wilcoxon-Mann–Whitney test for not normally distributed variables. Further, we compared categorical variables with Chi-Square or Fisher’s exact test. Spearman`s rank correlation was used to test dependence of nonparametric measures. Significance level was set at *p* = 0.05.

### Ethical approval

The study was approved by the ethics committee of Zürich (BASEC Nr. 2021-00285) and registered at clinical Trials.gov (NCT04816357).

## Results

Baseline characteristics of the 94 women with migraine and EM are demonstrated in Table 1. Mean age at migraine onset was 19.7 ± 8.4 years and mean age at menarche 12.7 ± 1.6 years. Migraine features, including the current frequency, MMF five years ago and at age younger than 20 years, migraine type and MIDAS score and grade are listed in Table 2. Altogether, the MMF was rather low at present and at age < 20 years, while it was substantially higher 5 years ago (*p* < 0.01 vs frequency now; *p* < 0.03 vs frequency at age < 20 years) (Table 2). Currently, 59% of the patients did not use any prophylactic agents, 37% used magnesium or riboflavin on a regular basis and 3.1% used Botox or b-blockers. Pain medication was prescribed from neurologists in 18.5%. and from GCPs in 35.1%. Another 35.1% of the participants bought the medication in the pharmacy without prescription and 11.3% received it from other sources. Migraine with aura (MA) had been reported form 41% of the women. Women with MA reported more frequently unilateral attacks (*p* < 0.045. In our setting MA was associated with younger age at migraine onset and with a history of medication overuse headache (MOH) (Table 3). No associations were found with ASRM stage (*p* < 0.37) or depth of infiltration of lesions (*p* < 0.45 for infiltration ≥ 3 cm).). MMF in the past did not differ between MA and MO patients. Women with a history of MOH suffered more often from more than 2 monthly attacks not now, but 5 years ago (*p* = 0.05), were more often triptan users (*p* = 0.05) and were more often using more than 3 days/months analgesics for dysmenorrhoea (*p* < 0.04) (Table 3). MOH was not associated with a personal history of depression (*p* < 0.25). Endometriosis ASRM score was not associated with MA, higher frequency of migraine or MOH. Patients with a history of MOH were also not affected by deeper infiltration of endometriosis lesions, what is known to be associated with more pain (*p* = 1.0 for infiltration ≥ 3 cm).

**Table 1:** Baseline and endometriosis characteristics

Patients’ characteristics:	Number of patients *n* (%)/mean ± standard deviation (min–max)	*N*
Age (years)	36.4 ± 7.6 (20–53)	94
Height (cm)	167.3 ± 6.0 (153–184)	94
Weight (kg)	65.2 ± 11.2 (40–120)	94
Body mass index (kg/m^2^)	23.3 ± 3.8 (17–43)	94
Age at first manifestation of migraine (years)	19.7 ± 8.4 (6–45)	88
Depression ever	37 (39.4)	94
Mean age at menarche	12.8 ± 1.6 (8–17)	93
Combined pill/patch/ring use at present	9 (9.6)	94
ASRM stage		94
1	25 (26.6)	
2	20 (21.3)	
3	21 (22.3)	
4	28 (29.8)	
Depth of infiltration > 3 cm	21 (22.3)	94
Dysmenorrhea score 2/3 yes	72 (77.4)	93
> 2 days of analgesics during menstruation	44 (46.8)	94

**Table 2:** Migraine features in women with comorbid endometriosis

Characteristics		Number of patients *n* (%)/mean ± SD (min–max)	*N*
Migraine with aura	(Yes)	39 (41.5)	94
Migraine onset < 17 years	(Yes)	39 (44.3)	94
Migraine frequency at age under 20:	< 1 migraine attack/month	50 (56.2)	89
	1 or 2 migraine attacks/month	18 (20.2)	89
	> 2 migraine attacks/month	21 (23.6)	89
Migraine frequency at age under 20	(Attacks/month)	1.6 ± 2.78 (0–15)	89
Migraine frequency five years ago:	(Attacks/month)	2.9 ± 4.64 (0–25)	90
Migraine frequency five years ago:	> 2 migraine attacks/month	28 (31.1)	90
Current migraine frequency:	(Attacks/month)	1.5 ± 2.65 (0–15)	93
Current migraine frequency:	> 2 migraine attacks/month	20 (21.5)	93
Mean duration of migraine attacks	(Hours)	16.6 ± 19.1 (0.5–72)	91
Ø migraine attack duration over 9 h	(Yes)	50 (54.9)	91
Status migraenosus ever	(Yes)	22 (23.4)	94
*Migraine symptoms*			
Unilateral migraine	(Yes)	63 (67.0)	94
Sensitivity to light	(Yes)	91 (96.8)	94
Sensitivity to noise	(Yes)	63 (67.0)	94
Nausea	(Yes)	83 (88.3)	94
Emesis	(Yes)	44 (46.8)	94
Triptan for acute migraine treatment	(Yes)	22 (23.7)	93
Ever MOH	(Yes)	8 (8.6)	93
MIDAS score		6.8 (13.0)	94
MIDAS grade (migraine disability assessment score)	Grade 1 (little or no disability)	60 (63.8)	94
	Grade 2 (mild disability)	15 (16.0)	94
	Grade 3 (moderate disability)	4 (4.3)	94
	Grade 4 (severe disability)	15 (16.0)	94

^a^Number of participants from 94 included patients

^b^severe pain: pain score 3 in a pain scale from 0 to 3

**Table 3:** Correlations for MA and MOH

MA	Correlation coefficient	*n*	95% confidence interval of odds ratio	*p*-value
First manifestation of migraine under 17 years old	0.331	88	0.124, 0.510	0.002
Current migraine frequency^a^	− 0.069	93	− 0.275, 0.143	0.511
Medication overuse headache	0.206	93	− 0.004, 0.398	0.048
*MOH*				
More than 2 attacks/month	0.033	92	− 0.179, 0.242	0.754
More than 2 attacks/month five years ago	0.220	89	0.006, 0.415	0.038
Triptan use	0.224	92	0.014, 0.415	0.032
More than 3 days/months analgesics for dysmenorrhoea	0.226	94	0.017, 0.416	0.029

^a^Migraine frequency in migraine days per month

## Discussion

There is little knowledge about the course and features of migraine in women who additionally suffer from comorbid endometriosis. Previous studies mainly focused on migraine prevalence in endometriosis patients, and cycle characteristics in patients with both, endometriosis and migraine [1, 2, 9, 23, 24]. Our sample included women in the middle of their reproductive years, recruited from an endometriosis clinic with an average BMI and typical age of migraine onset [25]. Mean age (36.4 years) was comparable with that in other endometriosis-migraine trials, but lower than in the CAMEO study, which focused on non-gynaecologic comorbidities. This needs consideration when interpreting the results [1, 2, 24, 26, 27]. Interestingly we found an MA rate of 41% in patients with endometriosis. MMF was low (1.5 attacks), potentially related to the selection of patients from a gynaecology outpatient clinic. It is surprising, that the reported MMF five years before the interview was significantly higher (2.9 attacks/month). Few women had ever experienced MOH (8.6%) and none of the participants suffered from chronic migraine at the time of the study. Mean age at menarche was 12.7 years and migraine onset before age 17 was reported by 44.3% of participants. Endometriosis ASRM score was not associated with MA, higher frequency of migraine or MOH. As a higher ASRM stage is not typically associated with more pelvic pain, we also tested for associations with depth of infiltration of the endometriosis lesions [28]. We also did not find an association here.

MMF in our trial was lower than in population-based studies [26, 27, 29]. We expected higher frequencies in women with two disabling pain comorbidities, especially as we see in our clinic for hormonal migraines rather patients highly affected by both conditions. The CAMEO study reported a MMF of 3.5 days for slightly older patients with other types of comorbidities in a huge sample representative for the US population [26]. For MMF at age < 20 years, our data are in accordance with findings from a diary -based study including migraine patients without endometriosis using combined hormonal contraceptives in a setting in Switzerland. In the latter study, MMF rose to 4.2 monthly attacks until mean age 26.5 years [30].

The decline of MMF during the reproductive years in our study is unusual [26, 31]. This decline compared to the frequency 5 years ago was probably not related to the new start of prophylactic agents, as the number of women using pharmaceutical products for headache prevention at the time of the interview was very low (3.2%). Therefore, we suggest that recent endometriosis surgery and treatment might play a pivotal role in the observed reduction. Improvement of one pain condition might exert a positive effect on the course of the second pain condition [17]. Long-term studies in migraine patients suffering from endometriosis are necessary to improve our understanding of the impact of endometriosis surgery and treatment on the course of migraine. Up to date, most studies addressing comorbidities of migraine focus on cardiovascular conditions, psychic conditions, and autoimmune disease but not on endometriosis [26, 32, 33].

Our patients reported a relatively long duration of migraine attacks with 16.6 h. In a recent Italian multicenter study, around 50% of the migraine patients suffered from migraine episodes lasting less than 24 h, while the mean duration in the CAMEO setting was 27.7 h [26, 34]. The duration of attacks might be related to insufficient response to acute medication. Only 18% of the patients in our setting had ever seen a neurologist to receive a prescription, and triptan use in this setting of an endometriosis clinic was rather low. Triptans would not reduce pain associated with endometriosis, what could contribute to the preferred use of other types of analgesics in patients with both conditions.

Two trials with patients recruited from a gynaecologic center did not report MA data and typical migraines features for the subgroup of patients with both, endometriosis and migraine patients [2, 35]. In comparison to the MA prevalence in population-based studies (5–6%), we found a much higher prevalence (41.5%) [34, 36, 37]. This finding is very much in line with the results from Ferrero, who also studied migraine features in more detail in women recruited from an endometriosis-clinic [38]. The strengths of our trial with a smaller sample size is the inclusion of only patients with surgically confirmed endometriosis and collection of migraine data not with a questionnaire but in interviews performed by neurologists. Ferrero et al. found a MA prevalence of 35% and a slightly higher migraine frequency with 44.7 attacks/year [38]. The higher MMF in this trial, performed more than 15 years ago, could be related to the high percentage of combined hormonal contraceptive (CHC) users (25.6%). Today it is better known among neurologists and gynaecologists, that CHCs should not be used in MA patients. In addition, CHC use for endometriosis treatment is less common. Considering the high genetic background with sex hormone receptor polymorphisms in endometriosis-migraine patients, we suggest that we have a special subgroup of migraineurs, with different migraine features, especially more frequent aura [3, 4]. Factors associated with MA in our trial were migraine onset at an earlier age ($\le$ 17 years) and a history of MOH. Earlier age at migraine onset might also be an indicator for a stronger genetic background. The pathophysiology of MA is by far not complete understood. One of the leading theories is that aura phenomena are linked to cortical spreading depression (CSD). Hormone fluctuations during the menstrual cycle, especially estrogen fluctuations might play a role in the development of CSD [39]. Endometriosis lesions may release estradiol. Sandweiss et al. found, that rats, after a 17-β-estradiol injection, developed significantly more CSD episodes over a 12-h recording period. Pre-administration of an estrogen receptor antagonist blocked CSD events and pain behaviors [40]. However, the low amounts of estrogen released locally from endometriosis lesions are minimal in comparison to the cyclic estrogen production in the ovaries. Endometriosis patients have regular cycles and do not differ from other healthy young women with regard to estrogen levels or fluctuations. Therefore, it seems unlikely, that the high prevalence of aura in endometriosis patients is generated from the estrogen release from endometriosis lesions.

None of our patients suffered from chronic migraine, which would have an expected prevalence in the female population of around 1.3% and might be even higher in women with comorbidities [26, 41]. We cannot exclude that with age some of our participants might develop chronic migraine in the future. In a small sample of endometriosis patients recruited from a tertiary headache center who were of similar age, Tietjen et al. found chronic migraine in 40% of women [35]. The reason for such differences is unclear but might be explained with differing recruitment-strategies.

Chronic pain conditions, more severe headache, psychic conditions and triptan use have been shown to be associated with a higher risk or medication overuse headaches (MOH) [32, 42–44]. MOH prevalence in the general population of migraineurs is 1–2%. It was 11.9% in a study including patients with non-gynaecologic comorbidities and much higher in trials conducted in headache clinics (50–72%) [26, 42, 45–47]. In our trial 8.6% of the participants reported to have ever experienced MOH. Surprising for us in this setting of women with two disabling pain conditions was, that at present none of the participants suffered from MOH. Special features of a history of MOH in our trial include more migraine attacks 5 years ago, migraine with aura, use of triptans (OR 4.9) and use of more pain killers to treat pelvic pain during the menstrual bleeding (Table 3). Use of pain killers for other conditions than migraine may contribute to the risk of developing MOH [48]. Again, we raise the theory, that the low MOH rate could be related to the improvement of endometriosis pain after surgery and treatment. Future prospective studies should focus on this point. The location for recruitment, i.e. headache clinic, gynaecologic setting, or general population, has to be taken into account when comparing results with other studies.

MIDAS score in our trial did not differ substantially from the CAMEO study (6.0 and 6.8). A MIDAS grade indicating moderate or severe disability in 20% of the women is of concern and reflects severe suffering in a subgroup of our sample.

Altogether, we observed in contrast to the normal course of migraine in women during the reproductive years a decrease in migraine frequency in our sample. If this improvement is causally related to endometriosis surgery, has to be investigated in further prospective studies. We confirm that the prevalence of MA is higher than expected from the general population. Migraine with aura, early migraine onset and episodic migraine have been shown to be associated with a positive family history [18, 49]. Therefore and based on the knowledge that endometriosis-migraine patients share common sex-hormone specific polymorphisms, we postulate a potential shared genetic background as reason for the high prevalence of MA. In comparison to studies with endometriosis-migraine patients recruited from headache clinics, we found much lower MMF, a lower MOH rate and no patients with chronic migraine. This might indicate that there are two subtypes and phenotypes of women with both conditions. On the one hand, those with endometriosis pain as the major burden and less migraines and on the other hand patients with migraine as the major burden, who would suffer from a higher MMF and more often from chronic migraines.

### Strength and limitations

Migraine features might differ between patients recruited from a gynaecologic clinic or a headache center. Our results apply to women who visited a specialised endometriosis clinic. Women recruited from a gynaecologic center with endometriosis pain as their major burden might suffer from less severe migraines than those recruited at headache centers. Only a subset had ever consulted a neurologist for their headache problem. Endometriosis was the more disabling condition for our participants. A limitation of this trial is the lack of a control group. Furthermore, we cannot exclude that there might be some recall bias related to interview data addressing events very long ago like age at menarche or migraine onset. Strength of the study include the sample size and the collection of migraine data through personal telephone interviews performed by specialists. In addition, the diagnosis of endometriosis was histologic confirmed and localization and ASRM score provided detailed information on the stage and phenotype of the condition.

## Conclusion

We conclude that the comorbidity of endometriosis maybe linked to MA, while MMF is rather low. Further studies are needed to explore, if the observed decrease in MMF at present in comparison to MMF 5 years ago, can be attributed to recent endometriosis surgery. ASRM score was not associated with any of the migraine features, nor was deep infiltration of the lesions.

## Data Availability

The datasets used and/or analysed during the current study are available from the corresponding author on reasonable request.

## Abbreviations

MO: Migraine without aura
MA: Migraine with aura
MO: Migraine without aura
MOH: Medication overuse headache
MMF: Monthly migraine frequency
ASRM: American society for reproductive medicine
